# Efficacy and Pharmacological Mechanism of *Poria cocos*-Based Formulas Combined With Chemotherapy for Ovarian Cancer: A Integrated Systems Pharmacology Study

**DOI:** 10.3389/fphar.2022.788810

**Published:** 2022-03-21

**Authors:** Xinya Peng, Congchao Jia, Hao Chi, Pengyu Wang, Hu Fu, Yunyue Li, Qin Wang

**Affiliations:** ^1^ Clinical Medical College, Southwest Medical University, Luzhou, China; ^2^ Department of Laboratory Medicine, Chengdu First People’s Hospital, Chengdu, China; ^3^ Queen Mary College, Medical School of Nanchang University, Nanchang, China; ^4^ Sichuan Treatment Center for Gynaecologic and Breast Diseases (Gynaecology), Affiliated Hospital of Southwest Medical University, Luzhou, China

**Keywords:** poria cocos, chemotherapy, ovarian cancer, meta-analysis, network pharmacology, molecular docking, PI3K/AKT signal pathway

## Abstract

Previous studies have shown that *Poria cocos*-based formulas combined with chemotherapy can improve the quality of life of ovarian cancer patients. However, the results are still controversial. We systematically searched the literature from eight databases to evaluate the efficacy and safety of *Poria cocos*-based formulas in combination with paclitaxel-carboplatin in treating ovarian cancer (OC). Subsequently, network pharmacology, molecular docking and cell experiments were performed to further verify the underlying molecular mechanism. Thirteen randomized controlled trials, including 922 patients with OC, were enrolled in the study. The results indicated that *Poria cocos*-based compounds combined with paclitaxel-carboplatin significantly improved patients’ tumor response rate, traditional Chinese medicine syndrome score, Karnofsky Performance Scale, physical and social function, and reduced side effects of chemotherapy compared to the paclitaxel-carboplatin alone. According to the network pharmacological analysis, tumulosic acid were the most bioactive compounds of *Poria cocos*. BCL2L1 is highly expressed in OC and is associated with a worse prognosis which could become potential drug target. Functional enrichment analysis suggested that the anti-OC effect of *Poria cocos* may be related to PI3K-Akt signaling pathway. The molecular docking results indicated that tumulosic acid might inhibit OC by regulating BCL2L1. Vitro experiment confirmed tumulosic acid that induced cell apoptosis by modulating PI3K/AKT signaling and BCL2L1. Our study may provide a clinical basis and theoretical rationale for combining *Poria cocos*-based formulas with chemotherapy for OC. In addition, the integrated pharmacological strategy proposed in our study provides an excellent example for exploring the mechanism of complex formulas.

## Introduction

Ovarian cancer (OC) is the seventh most common cancer among women worldwide and the eighth leading cause of cancer death, and the second most common cause of gynecologic cancer death (after cervical cancer), with a 5-years survival rate of less than 46% and an increasing rate of diagnosis each year (Lheureux et al., 2019a; Lheureux et al., 2019b). Since the ovaries are located deep in the pelvis, which is a hidden position, about 60–70% of patients were diagnosed in the advanced stage of OC due to a lack of early obvious or very particular symptoms (Ebell et al., 2016; Orr and Edwards 2018). Currently, intravenous administration of carboplatin and paclitaxel every 3 weeks is the standard first-line chemotherapy drug treatment for advanced-stage epithelial OC (Karam et al., 2017). However, the method generated several side effects, especially nausea and vomiting, bone marrow suppression, muscle pain, peripheral neuropathy, and hair loss (Lheureux et al., 2019a). Therefore, we require the continued search for complementary treatments to alleviate toxic effects.

Traditional Chinese Medicine (TCM) is a treasure-house that has shown miraculous clinical effects. Youyou Tu discovered artemisinin from TCM, saving millions of lives, reducing the mortality rate of malaria in Africa by 58%, and among children under five by 69% (malERA Refresh Consultative Panel on Health Systems and Policy Research, 2017). As far as we know, Chinese medicine adjuvant treatment of cancer has become common worldwide (Maskarinec et al., 2000; Abdullah et al., 2003; Ferro et al., 2007). *Poria cocos*, known as “Fuling” in Chinese, is an edible medicinal mushroom belonging to the dry sclerotium of Polyporaceae *fungi*. It has more than 2,000 years of medical application history for its remarkable pharmaceutical effect (Wang et al., 2013). Some formulas based on *Poria cocos* were often utilized in adjuvant chemotherapy for OC in clinical practice (Ruan et al., 2018). However, there was some controversy that *Poria cocos* did not improve the quality of life (QOL) of OC patients according to a high-quality randomized controlled trial (RCT) (Chan et al., 2011). Accordingly, we used a meta-analysis to assess the evidence for the efficacy of *Poria cocos* compound combined with carboplatin-paclitaxel chemotherapy in OC treatment.

Meta-analysis is a statistical method that combines different research results on the same topic and plays a vital role in medical research and clinical decision-making (Lee 2018). It is well known that meta-analysis pays more attention to the synthesis of clinical data, while ignoring the exploration of basic mechanisms. In recent years, along with the rapid progress in bioinformatics, network pharmacology has emerged as a holistic and efficient tool to decode the underlying mechanisms of multitarget treatments by analyzing various networks of complex and multilevel interactions (Li X. et al., 2020). Therefore, in order to investigate the mechanism and pathway of action of *Poria cocos* on OC, we used network pharmacology with molecular docking techniques and vitro validation for further exploration.

## Materials and Methods

### Meta-Analysis

#### Registration

Our study was registered in the International Platform of Registered Systematic Review and Meta-analysis Protocols with the registration number 202180060.

#### Search Strategy

We systematically searched PubMed, EMBASE, Cochrane Library, Web of Science, Chinese Science and Technology Journals (CQVIP), China Academic Journals (CNKI), Wanfang, and Chinese Biomedical Literature database to compare the efficacy of *Poria cocos*-based formula and chemotherapy, or with chemotherapy alone in the treatment of OC. The Medical Subject Heading (MeSH) terms and entry terms used to search literature were as follows: “*Wolfiporia* or *Poria cocos* OR Fuling OR Medicine, Chinese Traditional OR Traditional Chinese Medicine OR Traditional medicine” and “Ovarian Neoplasms OR Ovarian Cancer OR Ovary Neoplasms OR Ovary Cancer OR Cancer of Ovary”. The detailed search strategies are displayed in Supplementary text 1. All articles were published before 20 July 2021, and no restriction on language was applied.

#### Criteria for Inclusion and Exclusion

Studies that met the following PICOS criteria were included: 1) participants: OC patients; 2) intervention: *Poria cocos*-based formulas and carboplatin-paclitaxel; 3) comparator: carboplatin-paclitaxel; 4) outcomes: tumor response rate (TRR), TCM syndrome score, Karnofsky Performance Scale (KPS), quality of life (QOL), side effects; 5) study design: RCT.

Exclusion criteria: non-RCTs, animals and cells experiment, editorials, letters, meta-analysis, expert opinions, abstracts, case reports and reviews without original data, and studies lacking control groups. In addition, studies and data according to the following situation were also excluded: Raw data cannot be extracted; the outcomes of patients were not reported; studies from uniform institutions with identical data.

#### Data Extraction

All variables included in the study were extracted by two reviewers (Xinya Peng and Congchao Jia) independently, including the first author, year of publication, sample sizes of treatment group and control group, tumor stage, intervention, TCM duration, and clinical outcome indicators. Two reviewers discussed differences. When differences cannot be resolved, the third reviewer (Hao Chi) shall solve them.

#### Quality Assessment and Risk of Bias

Two investigators independently evaluated the risk of bias in RCTs using the Cochrane risk of bias tool, and a third reviewer resolved differences if necessary. The risk of bias tool, which is one of the most comprehensive approaches to assessing the potential for bias, covers six domains of bias: selection bias, performance bias, detection bias, attrition bias, reporting bias, and other biases (Higgins et al., 2011). Low, high, and unclear risk of bias represent that the study met the criteria, did not meet the criteria, and was unable to determine.

#### Statistics Analysis

Cochrane Review Manager software (RevMan, version 5.4) and STATA software (version 16.0) were utilized for statistical analysis. For dichotomous outcomes, results were expressed as a risk ratio (RR) with 95% confidence intervals (CI). The mean difference (MD) for continuous scales was used to assess treatment effects or the standardized mean difference (SMD) if different scales were used. Graded outcome data was analyzed by Generic Inverse Variance. The meaning of HR values was determined according to different outcome indicators. A *p* < 0.05 was considered statistically significant. Heterogeneity was assessed using the Q test and I^2^ statistics. A chi-squared *p* < 0.1 or an I^2^ statistic >50% was regarded as heterogeneity. When heterogeneity existed, we performed a sensitivity analysis to remove research with a higher risk of bias or used a random-effect model for data pooling; otherwise, a fixed-effects model can ensure the robustness of the model chosen and susceptibility to outliers was adopted. Forest plots indicated the results of the meta-analysis and a funnel plot was utilized to assess publication bias.

### Network Pharmacology Research

#### Identification of Ingredients and Targets of Poria cocos

The ingredients of *Poria cocos* come from two databases: Traditional Chinese Medicine database and Analysis Platform (TCMSP, https://tcmsp-e.com/, Retrieval date: 16 August 2021) and the Encyclopedia of Traditional Chinese Medicine (ETCM, http://www.tcmip.cn/ETCM/, Retrieval date: 16 August 2021). TCMSP is a systematic pharmacology database that brings together drug findings from previous herbal experiments. The database contains pharmacochemistry, pharmacokinetics and toxicity profiles, drug similarities and targets, related diseases, and interaction networks. Notably, the database can be used to reveal the active ingredients in Chinese medicine and their targeted cellular pathways (Ru et al., 2014; Li C. et al., 2020). ETCM includes comprehensive and standardized information for the commonly used herbs and formulas of TCM and their ingredients. To facilitate functional and mechanistic studies of TCM, ETCM provides predicted target genes of TCM ingredients, herbs, and formulas. A systematic analysis function is also developed in ETCM, which allows users to explore the relationships or build networks among TCM herbs, formulas, ingredients, gene targets, and related pathways or diseases (Xu et al., 2019). In addition, to obtain the effective ingredients of *Poria cocos* more comprehensively, we searched in PubMed, embase, Web of Science and supplemented the active ingredients with anti-cancer effects. We supplement the results of a recent study based on UHPLC-MS/MS (Zhu et al., 2018). After all collected components were combined and deduplicated, the Swiss target prediction (http://www.swisstargetprediction.ch/, Retrieval date: 16 August 2021) was used for target fishing. Fishing target is a calculation method that can use target structure information and biological database data to identify biological targets of active compounds.

#### Identification of Related Targets for OC

The related targets for OC came from three databases: 1) The GeneCards database (https://www.genecards.org/, Retrieval date: 20 August 2021). GeneCards is the human gene compendium that enables researchers to effectively navigate and inter-relate the wide Universe of human genes, diseases, variants, proteins, cells, and biological pathways (Stelzer et al., 2016). According to previous experience, we selected relevance score >10 as the threshold screening target (Shang et al., 2021; Jia et al., 2021). 2) The Open Targets Platform (OTP, https://platform.opentargets.org/, Retrieval date: 20 August 2021). The Open Targets Platform is a comprehensive tool that supports systematic identification and prioritization of potential therapeutic drug targets (Ochoa et al., 2021). In this study, to obtain more accurate results, we chose the overall association score >0.25 as the threshold. 3) DisGeNET (https://www.disgenet.org/, Retrieval date: 20 August 2021) DisGeNET is a discovery platform containing one of the largest publicly available collections of genes and variants associated with human diseases. The current version of DisGeNET (version 7.0) contains 1,134,942 gene-disease associations, between 21,671 genes and 30,170 diseases, disorders, traits, and clinical or abnormal human phenotypes, and 369,554 variant-disease associations, between 194,515 variants and 14,155 diseases, traits, and phenotypes (Piñero et al., 2020). Then, the targets from the three databases were merged and deduplicated. To clarify the target of *Poria cocos* in the treatment of OC, we intersected the target of *Poria cocos* with that of OC.

#### Construction of Protein-Protein Interaction (PPI) Network

The intersection targets were imported into the STRING platform (https://string-db.org/, Retrieval date: 24 August 2021) to construct a PPI network. STRING is a search tool for retrieving interacting genes/proteins, which is applied for predicting the PPI network and detecting the possible relationships (Szklarczyk et al., 2021). The confidence level is set to high confidence (0.7), and the others keep the default settings. Then perform network topology analysis on the PPI network to determine the core target, and the core targets of *Poria cocos* in OC treatment with degree ≥2 × median and closeness centrality ≥0.45 were selected.

#### Target Further Validation

UCSC Xena (https://xenabrowser.net/datapages/, Retrieval date: 15 September 2021) (Vivian et al., 2017), an online tool for exploring gene expression, clinical and phenotypic data, obtained 33 cancer mutations from TCGA data, RNA sequence data, and clinical data. We downloaded UCSC XENA RNAseq data in TPM format for TCGA and GTEx processed uniformly by the Toil process. Moreover, we extracted RNA-seq data and clinical information from 495 cases of OC projects. RNAseq data in TPM (transcripts per million reads) format and log2-transformed were used to compare samples. Visualization was performed by R package (www.R-project.org) (Dessau and Pipper 2008) (Ggplot [v.3.3.3]). we also used the above data to conduct ROC analysis of key targets through R package [3.6.3 version]: pPOC package [1.17.0.1 version] and ggplot2 package [3.3.3 version]. The database is publicly open access and availability and therefore does not require approval from the local ethics committee.

The UALCAN database (http://ualcan.path.uab.edu/analysis-prot.html, Retrieval date: 15 September 2021) (Chandrashekar et al., 2017) is an interactive portal for in-depth analysis of TCGA gene expression data which we can use for protein expression analysis of the CPTAC (Clinical Proteomic Tumor Analysis Consortium) dataset.

GEPIA2 database (http://gepia2.cancer-pku.cn/, Retrieval date: 15 September 2021) (Tang et al., 2019) is an online site that analyzes RNA-seq expression data from 9,736 tumor samples and 8,587 normal samples from TCGA and GTEx projects. We used the “Survival Analysis” module of GEPIA2 to obtain the OS (Overall Survival) significance map data of key genes in TCGA-OC. Cutoff-high (50%) and Cutoff-low (50%) values were used as expression thresholds, and the groups were divided into high and low expression groups. A log-rank test was used for hypothesis testing.

The Human Protein Atlas (https://www.proteinatlas.org/, Retrieval date: 15 September 2021) (Uhlén et al., 2015), dedicated to providing information on the tissue and cellular distribution of all 24,000 human proteins, provides extensive proteomic and transcriptomic information on diverse human samples, including cell, tissue and pathology profiles. Therefore, we qualitatively analyze the cellular location of target genes and the differential expression of proteins.

#### Functional Enrichment Analysis

To elucidate the potential molecular mechanism of *Poria cocos* against OC, Gene ontology (Go) and Kyoto Encyclopedia of Genes and Genomes (KEGG) analysis were performed on the core targets based on the DAVID database (https://david.ncifcrf.gov/, Retrieval date: 25 August 2021).

#### Network Construction

In this study, three networks were constructed. 1) The PPI network. The construction method was as described above. 2) The herb-ingredient-target-disease (H-I-T-D) network. It was constructed based on the interaction between herb, ingredient, target, and disease and can efficiently reflect their relationship. 3) The herb-target-pathway (H-T-P) network. It was constructed based on the result of the KEGG analysis.

### Molecular Docking

In order to further clarify the interaction between active ingredients and key targets, we carried out molecular docking. We got the 3D structure of the key targets in the PDB database (https://www.rcsb.org/, Date retrieved: 9 September 2021). The screening criteria were: 1) The biological source of protein structure is human; 2) The resolution of protein crystals is less than 2.5 Å; 3) The PH value is as close as possible to the normal physiological range of the human body; 4) The sequence of the protein conformation is as complete as possible, combined there must be small molecule ligand information in the body. The 2D or 3D structure of the molecular ligand was downloaded from the PubChem database (https://pubchem.ncbi.nlm.nih.gov/, Date retrieved: 9 September 2021). Small molecule ligands were removed from the protein receptor using PyMOL 2.4.0, protein dehydration and hydrogenation were performed using Autodock, and the charges of the small molecules were calculated. The receptor protein docking site parameters were set to the smallest box that could contain the entire protein. Finally, the receptor was docked to the corresponding small molecule ligand using Autodock Vina. The visualization of the docking process is also done using PyMOL 2.4.0.

### Cell Experiments

#### Cell Culture

SKOV3 cells, a human ovarian cancer cell line, were purchased from ICell Bioscience Inc. (Shanghai, China). The cells were cultured in RPMI-1640 medium containing 10% fetal bovine serum, 100 μg/ml ampicillin, and 100 μg/ml streptomycin, at 37°C with 5% CO2 in a cell culture incubator.

#### Flow Cytometry Analysis for Apoptosis

SKOV3 cells were incubated in 6-well plates at 37 °C and treated with tumulosic acid at different concentrations (0, 30, 50, and 80 μg/ml) for 48 h. Subsequently, cell apoptosis was measured through flow cytometry with the Annexin V-fluorescein isothiocyanate/propidium iodide (Annexin V-FITC/PI) apoptosis detection kit (Sanjian Biotechnology Co. Ltd, Tianjin, China; AO 2001-02P-G) according to the manufacturer instruction. Briefly, treated cells were collected and washed twice with cold PBS, resuspended in 300 μl 1× binding buffer with 5 μl Annexin V-FITC and 5 μL PI staining solution, and incubated for 15 min in the dark at room temperature before using flow cytometry.

#### Western Blotting Assay

SKOV3 cells were seeded onto 6-well plates and treated with tumulosic acid or poricoic acid A respectively (0, 30, 50, and 80 μg/ml). Total protein was obtained and centrifuged at 13,000 g and 4 °C for 5 min. A bicinchoninic acid assay kit (ASPEN Biotechnology Co. Ltd, Wuhan, China; AS1086) was employed to detect the concentration of total protein. Proteins were separated using SDS-PAGE (ASPEN Biotechnology Co. Ltd, Wuhan, China; AS1012) and the separated proteins were transferred to a PVDF membrane at 300 mA. The membrane was blocked with 5% skimmed milk in PBS plus 0.1% Tween 20 (PBST) for 60 min, incubated with primary antibodies overnight at 4 °C: PI3K (CST, #4255), *p*-AKT (CST, #4060), AKT (CST, #9272), BCL2L1 (CST, #2764), BCL-2 (Abcam, ab196495) and GAPDH (Abcam, ab181602). Then incubated with goat anti-rabbit horseradish peroxidases (ASPEN Biotechnology Co. Ltd, Wuhan, China; 1:10,000, AS1107) for 30 min at room temperature. Finally, protein band development was performed. The film was scanned and archived, and the AlphaEaseFC software processing system analyzes the optical density values of the target band.

## Results

### Meta-Analysis

#### Baseline Study Characteristics

**FIGURE 1:**
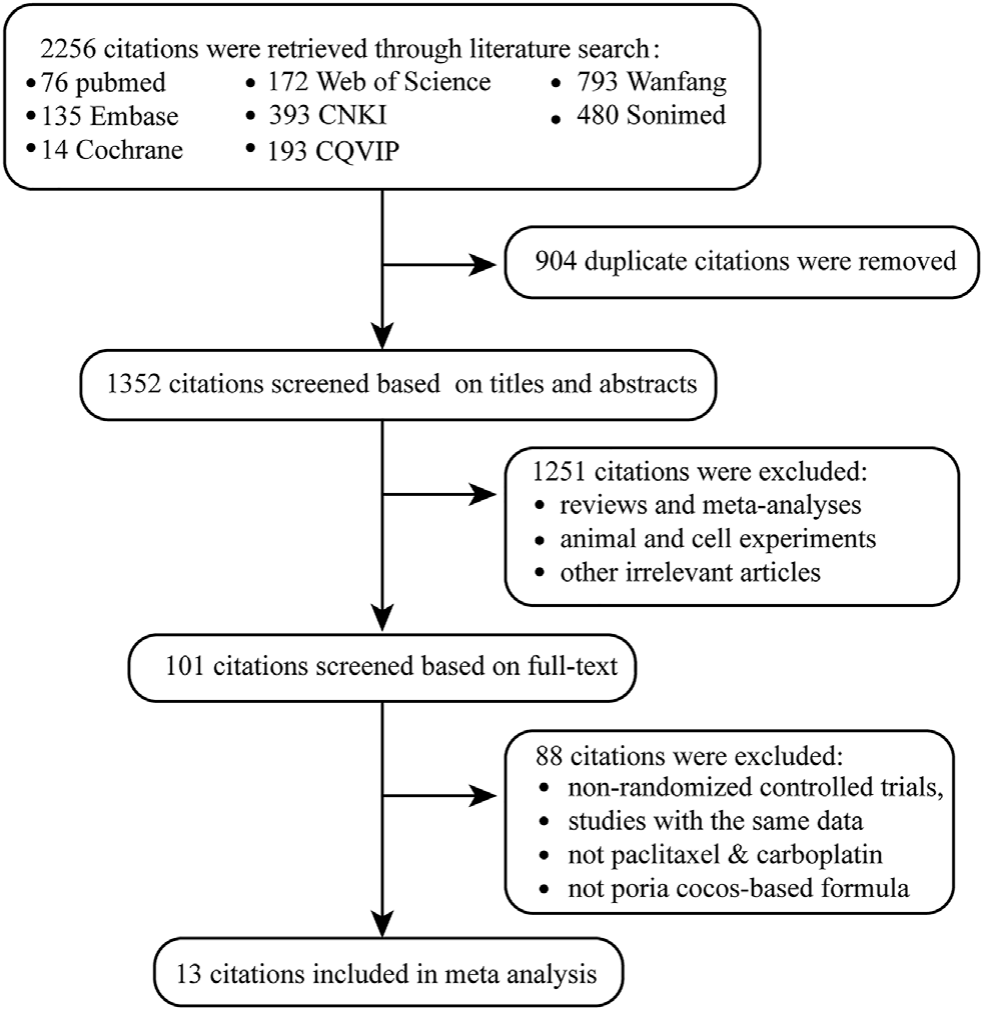
Flow diagram for selecting eligible studies.

A total of 2,256 citations were retrieved (Figure 1). After deleting 904 duplicate articles, 1,352 articles remained. We reviewed titles and abstracts, 144 reviews and meta-analyses, 479 animal and cell experiments, and 628 irrelevant articles. Eighty-eight articles were excluded for the following reasons: non-RCTs, studies with the same data, chemotherapy was not paclitaxel combined with carboplatin, and traditional Chinese medicine treatment was not *Poria cocos*-based formulas. Ultimately, 13 studies that contained 922 patients with OC were included in our meta-analysis.

Table 1 indicates the basic characteristics of all included articles. These studies were published between 2008 and 2020. There were 464 patients with OC receiving *Poria cocos*-based formulas combination with paclitaxel-carboplatin. In comparison, the other 458 patients were treated with paclitaxel-carboplatin alone. According to the Cochrane risk of bias tool, the risk of bias assessment revealed that the included studies were high quality (Supplementary Figure S1).

**TABLE 1:** Characteristics of the included studies.

Study	Treatment measures		Sample size (T/C)	Tumor stage	TCM duration	Outcomes
	Treatment group	Control group				
Kun (2010)	PTX + CBP plus Poria cocos-based formulas	PTX + CBP	25/25	I ∼ IV	9 weeks	B, C, D, E, F
Xiangyan (2017)	PTX + CBP plus Poria cocos-based formulas	PTX + CBP	42/42	NR	6–9 weeks	A, B, E
Tingzhen (2020)	PTX + CBP plus Poria cocos-based formulas	PTX + CBP	57/57	NR	6 weeks	A, E
Jinrong (2016)	PTX + CBP plus Poria cocos-based formulas	PTX + CBP	19/20	II, IV	6 weeks	B, D, E
Yue (2011)	PTX + CBP plus Poria cocos-based formulas	PTX + CBP	20/21	III, IV	NR	B, C, D, F
Fangfang (2019)	PTX + CBP plus Poria cocos-based formulas	PTX + CBP	44/44	I ∼ III	18 weeks	B, D, E, F
Yi (2013)	PTX + CBP plus Poria cocos-based formulas	PTX + CBP	19/19	NR	8 weeks	F
Yang (2008)	PTX + CBP plus Poria cocos-based formulas	PTX + CBP	25/23	I ∼ IV	6∼9 weeks	B, C, D, F
Xiaomao (2019)	PTX + CBP plus Poria cocos-based formulas	PTX + CBP	38/38	III, IV	2 months	A, D, E
Ruojia (2013)	PTX + CBP plus Poria cocos-based formulas	PTX + CBP	60/60	III, IV	18 weeks	B, D, F
Juan (2020)	PTX + CBP plus Poria cocos-based formulas	PTX + CBP	30/28	II ∼ IV	6 weeks	B, C, D, E, F
Chan et al. (2011)	PTX + CBP plus Poria cocos-based formulas	PTX + CBP	29/25	I ∼ IV	6cycle	B
Qinai (2015)	PTX + CBP plus Poria cocos-based formulas	PTX + CBP	31/31	III, IV	3 weeks	A, B, C, D, F

NR: not reported; A: TRR; B: QOL; C: TCM, syndrome score; D: side effects; E: tumor biomarker; F: immune cell. TRR, tumor response rate; QOL, quality of life; TCM, traditional Chinese medicine.

#### Effects of Intervention on TRR, TCM Syndrome Score, and KPS

**FIGURE 2:**
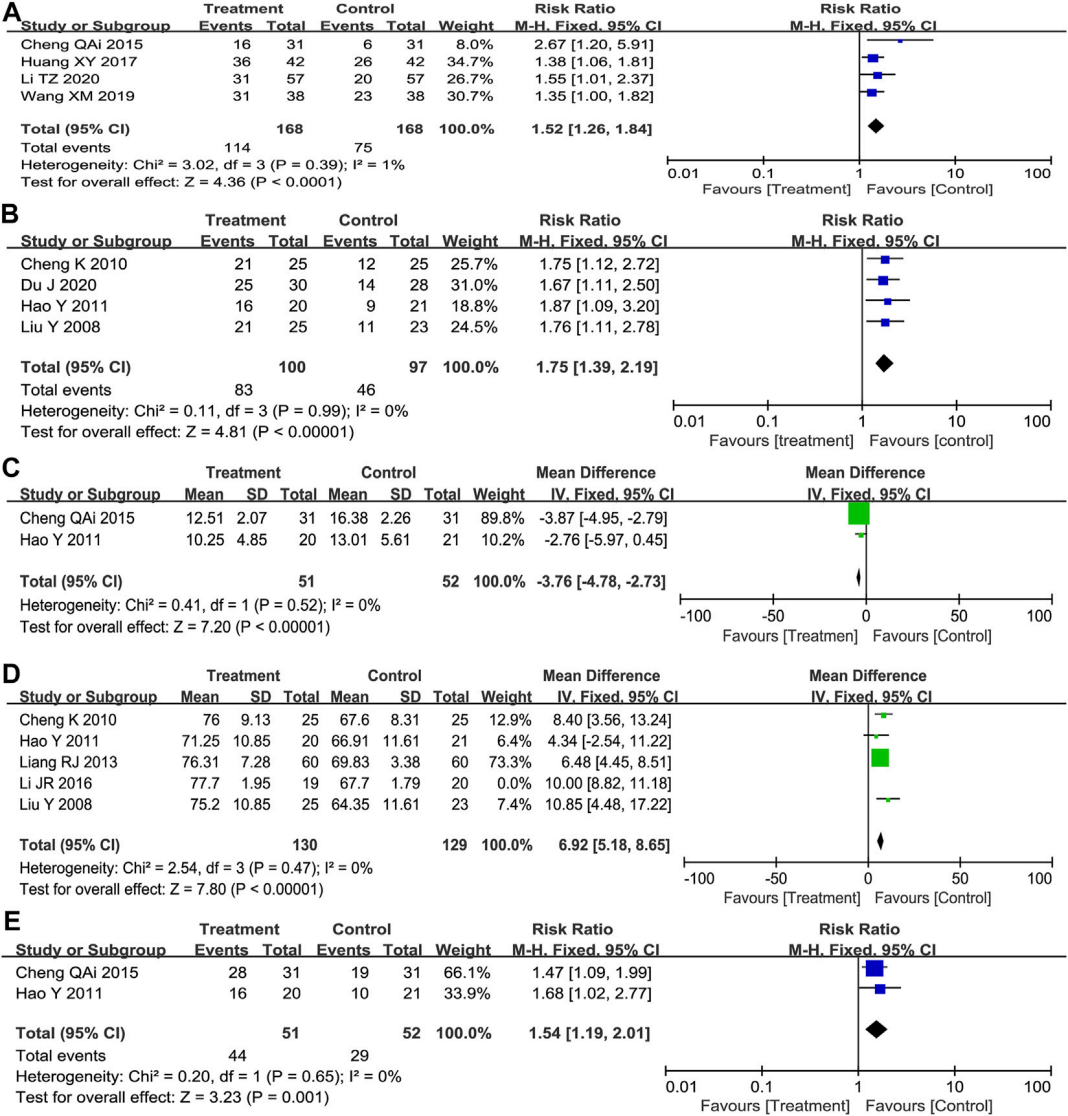
Forest plot displayed the results of the meta-analysis. **(A)** TRR. **(B)** TCM syndrome score for dichotomous outcomes. **(C)** TCM syndrome score for continuous scales. **(D)** KPS for continuous scales. **(E)** KPS for dichotomous outcomes. KPS, Karnofsky Performance Scale.

Tumor response criteria contain the complete response (CR), partial response (PR), stable disease (SD), and progressive disease (PD). CR plus PR was included as TRR. As shown in Figure 2A, we found that *Poria cocos*-based formulas combination with Paclitaxel-carboplatin significantly increased TRR compared with Paclitaxel-carboplatin (RR = 1.52; 95%CI:1.26–1.84; *p* < 0.001; I^2^ = 1%).

According to the common symptoms and signs of OC patients, including lower abdominal pain, abdominal distension, poor appetite, fatigue, emaciation etc, they were divided into three grades: mild, moderate, and severe. The TCM syndrome score was 1, 2, and 3 points, respectively. The proportional Integral (PI) was calculated using the following formulas:

$$PI=\frac{A-B}{C}\;$$

(A: total points before treatment; B: total points after treatment; C: total points before treatment×100%.) Significant effect: 70% ≤ PI < 100%; valid: 30% ≤ PI <70% and invalid, PI < 30%. The number of effective patients assessed in the four studies (RR = 1.75; 95%CI:1.39–2.19; *p* < 0.001; I^2^ = 0%) (Figure 2B) and two of these research that reported Mean ± SD of Symptom score (MD = 3.76; 95%CI: 4.78 to -2.73; *p* < 0.001; I^2^ = 0%) (Figure 2C) showed that chemotherapy combined with *Poria cocos*-based formulas could effectively reduce the clinical symptoms and signs of patients.

KPS was used to evaluate the physical status of patients. The fixed-effects model showed significant heterogeneity among the five included studies (*p* = 0.004, I^2^ = 74%), so sensitivity analysis was performed. After excluding one study that caused high heterogeneity, the results indicated that the combination of TCM formulas combined with chemotherapy was superior to the control group in the improvement rate of KPS score (MD = 6.92; 95%CI: 5.18–8.65; *p* < 0.001; I^2^ = 0%) (Figure 2D). Meanwhile, two trials assessing the number of improved patients (RR = 1.54; 95% CI: 1.19–2.01; *p* = 0.001, I^2^ = 0%) (Figure 2E) showed the same results.

#### Changes in QOL

**FIGURE 3:**
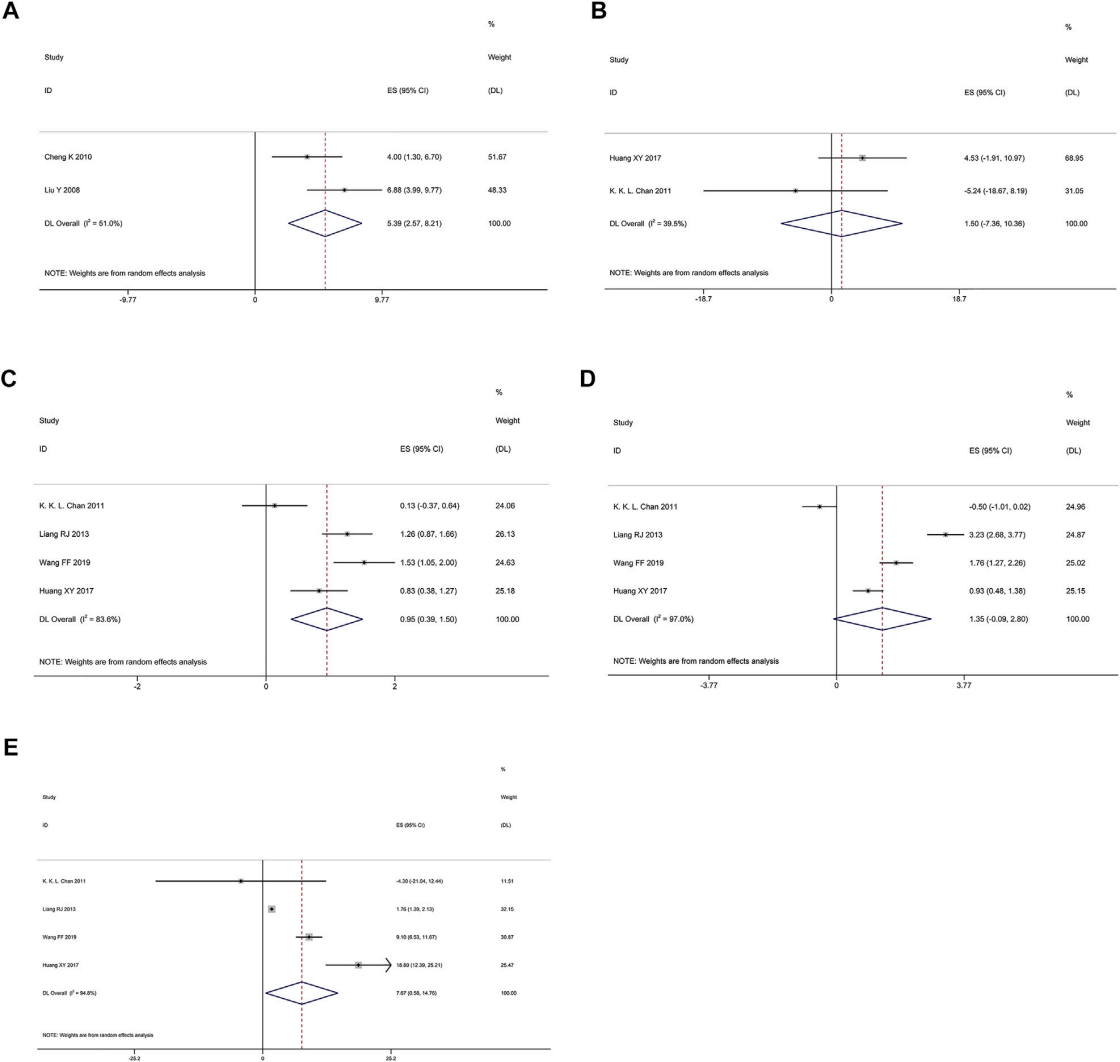
Random effects model. **(A)** QOL. **(B)** Cognitive function. **(C)** Physical function. **(D)** Emotional function. **(E)** Social function.

QOL was measured the specific questionnaire, including physical function, emotional function, cognitive function and social functional scales, etc. As shown in Figure 3, random effects model showed that *Poria cocos* formulas combined with chemotherapy could significantly improve the quality of life of patient (ES = 5.39; I^2^ = 51%), physical function (ES = 0.95; I^2^ = 83.6%) and social function (ES = 7.67; I^2^ = 94.8%). However, for emotional function (ES = 1.35; 95% CI: 0.09–2.80; I^2^ = 97%) and cognitive function (WMD = 2.70; 95% CI: −3.11 to 8.51; I^2^ = 39.5%; *p* = 0.199), there was no statistical significance.

#### Assessment of Side Effects

**FIGURE 4:**
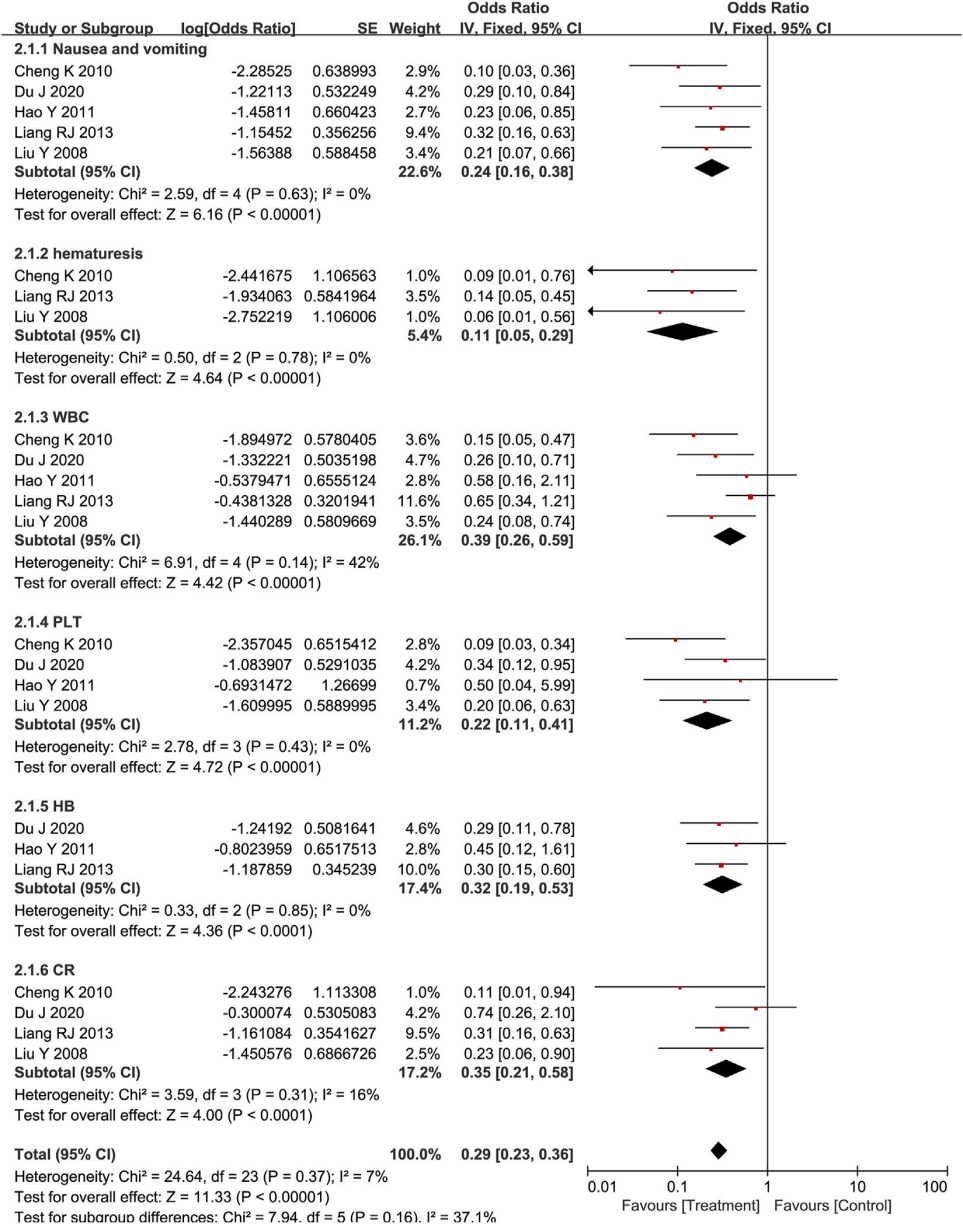
Graded outcome data of side effects.

Side effects included nausea and vomiting, hematemesis, myelosuppression (Reduction of white blood cells, platelets, and hemoglobin), and increases in creatinine. According to Standards for diagnosing and treating common tumors in China, the severity of side effects is divided into I-V. The higher the score, the more serious the adverse reaction. As shown in Figure 4, *Poria cocos*-based formulas with chemotherapy could significantly reduce the incidence of nausea and vomiting (OR = 0.24; CI:0.16–0.38; *p* < 0.001; I^2^ = 0%), hematemesis (OR = 0.11; CI:0.05–0.29; *p* < 0.001; I^2^ = 0%), reduction of white blood cells (OR = 0.39; CI:0.26–0.59; *p* < 0.001; I^2^ = 42%), platelets (OR = 0.22; CI:0.11–0.41; *p* < 0.001; I^2^ = 0%) and hemoglobin (OR = 0.32; CI:0.19–0.53; *p* < 0.001; I^2^ = 0%), increases in creatinine (OR = 0.35; CI:0.21–0.58; *p* < 0.001; I^2^ = 16%) and improve the corresponding symptoms. The funnel plot is symmetrical, indicating that there was no publication bias (Supplementary Figure S2).

### Network Pharmacology Research

#### The Ingredients and Targets of Poria cocos

Thirty-four ingredients were retrieved from TCMSP, and 32 ingredients were retrieved from ETCM. In addition, one ingredient was added based on literature retrieval. A total of 52 ingredients were obtained after combined weight removal. These ingredients and corresponding PubChem ID were shown in Supplementary Table S1. A total of 478 targets were derived after target fishing, which is shown in Supplementary Table S2.

#### The Related Targets of OC

**FIGURE 5:**
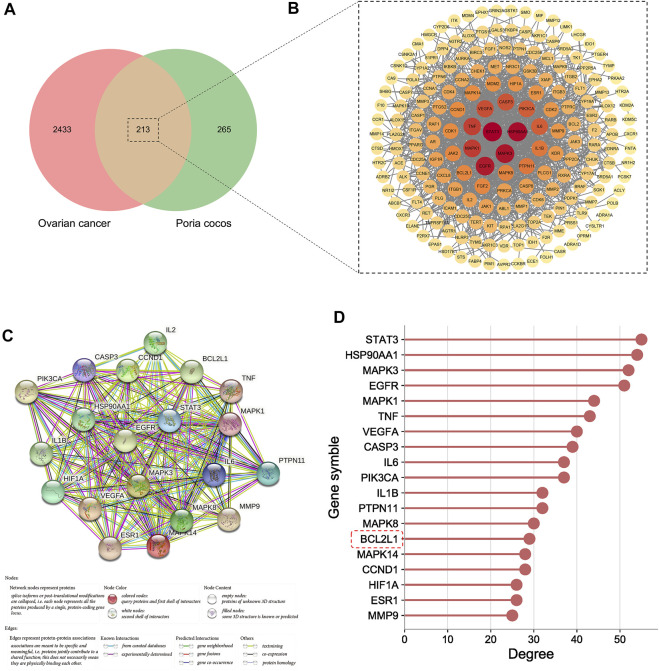
The process of core target screening. **(A)** The Venn diagram of *Poria cocos* and OC targets. **(B)** The PPI network of intersection targets. **(C)** The PPI network of core targets. **(D)** The degree value of core targets.

1,463, 915, 1,244 targets were first obtained from GeneCards, OTP, and DisGeNET, respectively, and then they were combined and deduplicated to obtain a total of 2,646 targets such as BCL2L1, TP53, ATM, and CDH1. The detailed information of these targets is shown in Supplementary Table S3. A total of 213 targets were obtained after taking the intersection of *Poria cocos* and OC targets (Figure 5A).

#### The PPI Network of Targets

The PPI network of intersection targets is shown in Figure 5B. According to the results of network topology analysis, 20 targets, including STAT3, and CASP3, BCL2L1 were identified as core targets. The PPI network of core targets was shown in Figure 5C, and the degree value of core targets was shown in Figure 5D.

#### Expression and Survival Analysis of BCL2L1

**FIGURE 6:**
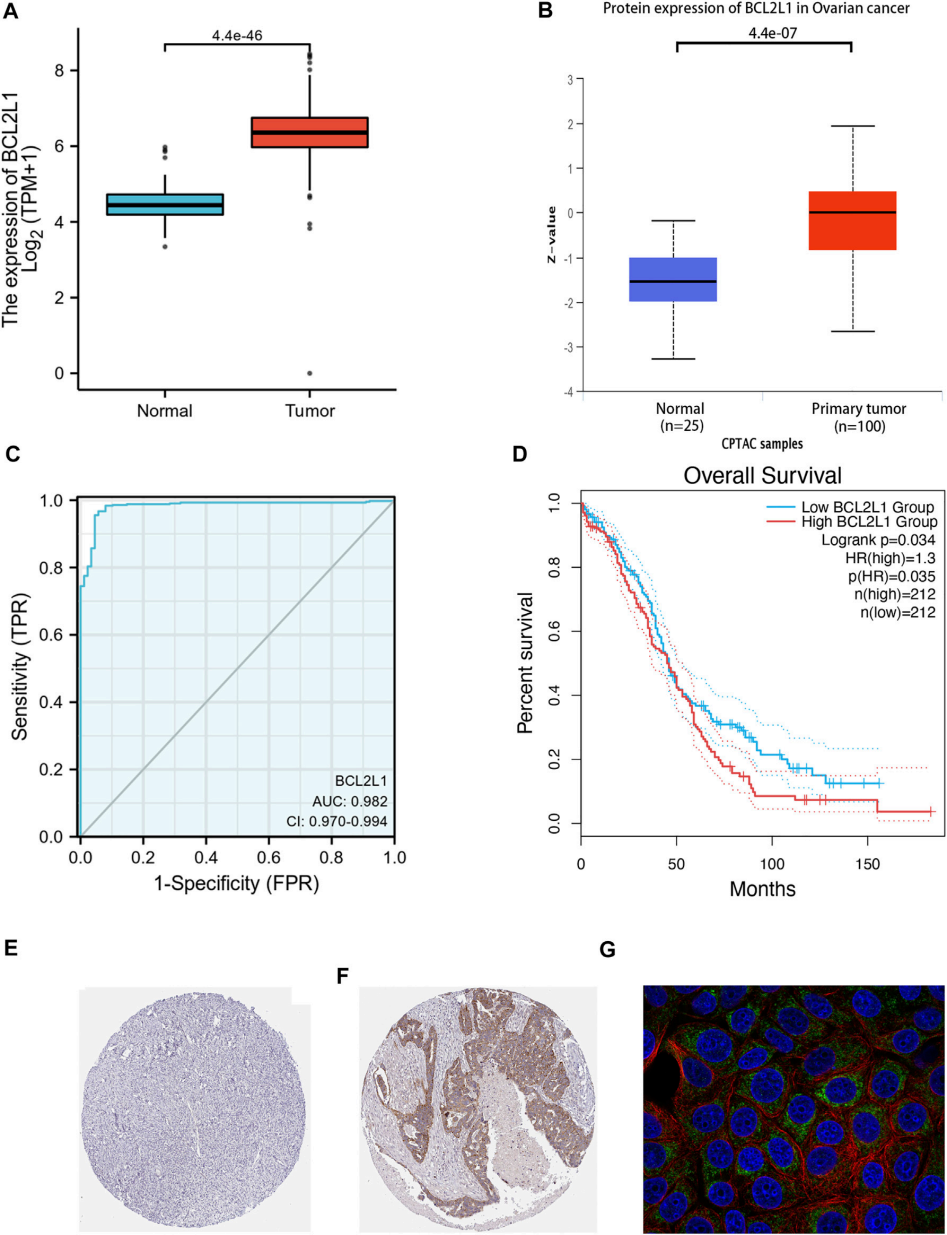
BCL2L1 is highly expressed in OC and correlates with poor patient prognosis. **(A)** Wilcoxon signed rank-sum test was used to analyze the difference in BCL2L1 expression between normal and OC samples of OC tissues in the assay GTEx. **(B)** Based on the CPTAC dataset, the expression levels of total BCL2L1 protein were analyzed between normal and primary tissues of OC. **(C)** ROC curves showed that BCL2L1 expression levels could effectively distinguish OC tissues from non-tumor tissues. The *x*-axis indicates the false positive rate and the *y*-axis indicates the true positive rate. **(D)** High BCL2L1 expression was correlated with poor overall survival prognosis in OC patients by GEPIA2 database analysis. **(E–F)** Human protein profiles showed higher BCL2L1 protein levels in OC tissues than in normal tissues (Antibody HPA035734). **(G)** Staining in the MCF-7 cell line revealed that BCL2L1 was localized to the mitochondria.

Survival analysis of the TCGA-OC dataset showed that high BCL2L1 expression was associated with poor overall survival prognosis (*p* = 0.035) (Figure 6D), while the other genes STAT3 (*p* = 0.33), HSP90AA1 (*p* = 0.94), MAPK3 (*p* = 0.28), EGFR (*p* = 0.51), MAPK1 (*p* = 0.3), TNF (*p* = 0.86), VEGFA (*p* = 0.86), CASP3 (*p* = 0.44), IL6 (*p* = 0.54), PIK3CA (*p* = 0.71), IL1B (*p* = 0.36), PTPN11 (*p* = 0.6), MAPK8 (*p* = 0.85), MAPK14 (*p* = 0.58), CCND1 (*p* = 0.12), HIF1A (*p* = 0.46), ESR1 (*p* = 0.29), MMP9 (*p* = 0.18) had no prognostic value (Supplementary Figure S3). Therefore, we selected BCL2L1 for further study. We obtained case data from the UCSC XENA database to analyze the expression of BCL2L1 in OC and compared the expression of BCL2L1 in 88 standard samples from the GTEx database and 427 OC samples from the TCGA database, and found that the expression level of BCL2L1 was significantly higher in OC tissues than in normal tissues (*p* < 0.001) (Figure 6A). The CPTAC database results showed that total BCL2L1 protein was expressed in the primary tissues of OC higher than normal tissues (*p* < 0.001) (Figure 6B). Also, the human protein atlas showed higher BCL2L1 protein levels in OC tissues than in normal tissues (Figures 6E,F). Furthermore, we used the subject operating characteristic (ROC) curve to analyze the effectiveness of BCL2L1 expression levels in distinguishing OC tissues from non-tumor tissues. The area under the curve (AUC) of BCL2L1 was 0.982, indicating that BCL2L1 can be an ideal biomarker to distinguish OC from non-tumor tissues (Figure 6C). Localization of the MCF-7 cell line revealed that BCL2L1 was mainly situated in mitochondria (Figure 6G).

#### Functional Enrichment Analysis of Core Targets

Based on the result of GO, there were 198 terms of biological processes (BP), including positive regulation of nitric oxide biosynthetic process, activation of MAPK activity, and negative regulation of the apoptotic process. There were 24 terms of molecular function (MF), such as nitric-oxide synthase regulator activity, growth factor activity, and histone deacetylase binding; there were 17 terms of cellular component (CC), such as cytosol, nucleoplasm, and cytoplasm. In addition, there were 101 pathways for the results of KEGG including PI3K/AKT signal pathway. The top 20 signaling pathways are listed in Supplementary Table S4.

#### Network Construction

**FIGURE 7:**
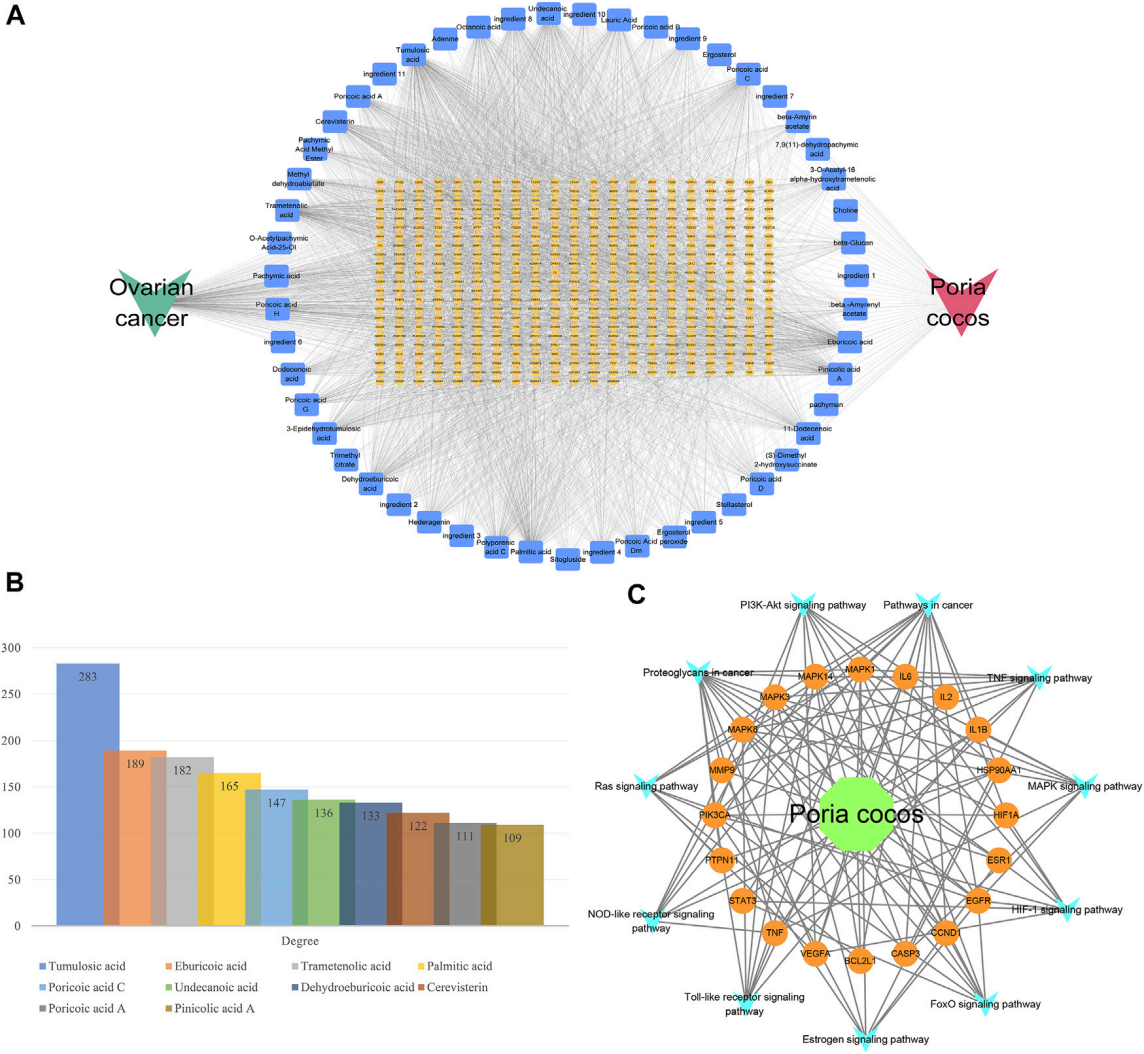
Network construction. **(A)** The H-I-T-D network. **(B)** The degree values of important nodes in network. **(C)** The H-T-P network.

The H-I-T-D network was shown in Figure 7A. As the names of some ingredients were too long to be displayed, they were denoted by “ingredient X,” and the specific correspondence was shown in Supplementary Table 4. The results of the network topology analysis of the H-I-T-D network are presented in Figure 7B. It suggested that tumulosic acid, eburicoic acid, and trametenolic acid play an important role in *Poria cocos* in treating OC. The H-T-P network was illustrated in Figure 7C, which elucidated the relationship between herb, targets, pathways and disease.

### Molecular Docking

**FIGURE 8:**
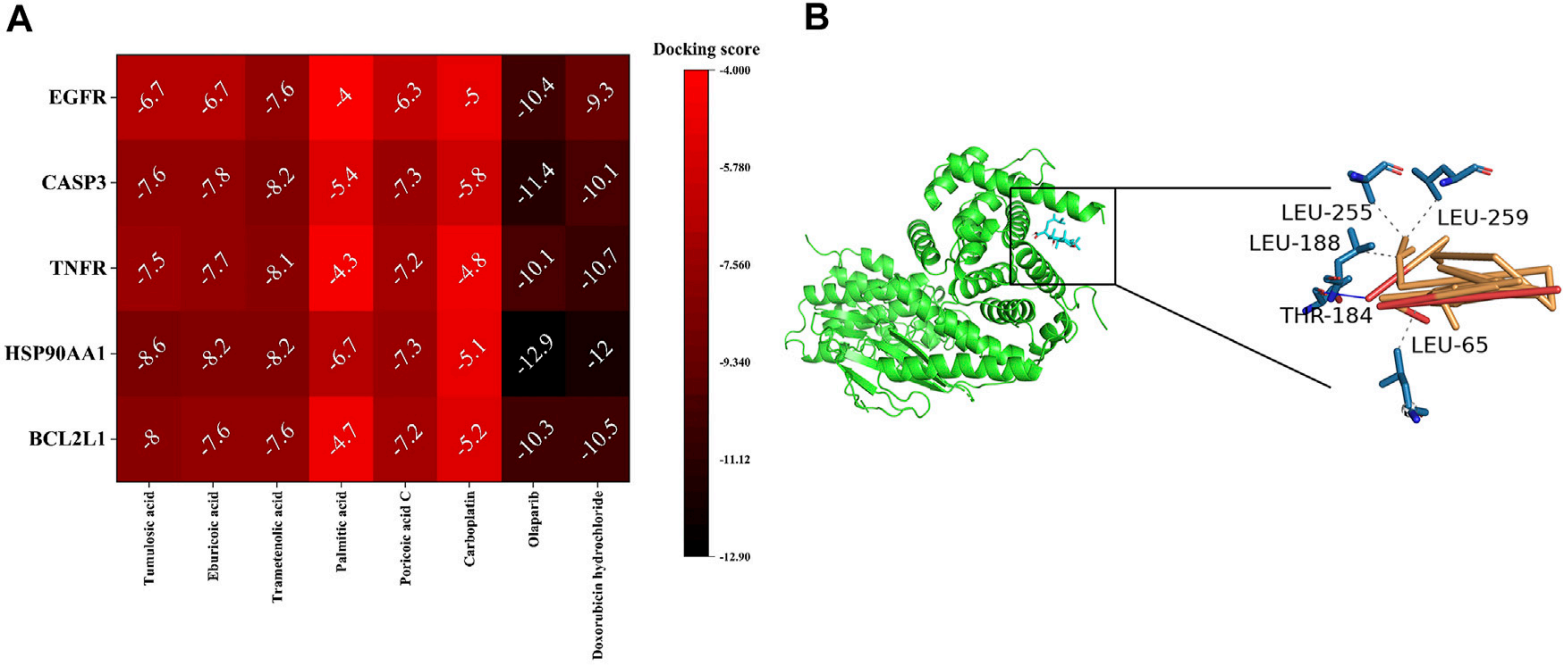
Ingredient-target molecular docking. **(A)** The docking score of Ingredient-target. **(B)** The docking diagram of tumulosic acid-BCL2L1. Dashed lines represent hydrogen bonds.

The docking score was used to indicate the affinity between the receptor and the ligand, and the smaller docking score represented a higher affinity. It was reported that a docking score less than -5 could be considered as a good binding between the receptor and the ligand (Zhou et al., 2016), but for more accuracy, we compared it with the clinically commonly used anti-OC drugs (carboplatin, Doxorubicin hydrochloride) as the standard control. The docking scores of ingredient-target are shown in Figure 8A. Molecular docking results showed that most of the ingredients and targets had an excellent affinity, suggesting that these components and targets played a vital role in treating OC by *Poria cocos*. In particular, the minor docking score between tumulosic acid and BCL2L1 suggested that tumulosic acid might inhibit OC by regulating BCL2L1 (Figure 8B).

### Validation *in Vitro*

#### Tumulosic Acid Promoted Apoptosis of SKOV3 Ovarian Cancer Cells

**FIGURE 9:**
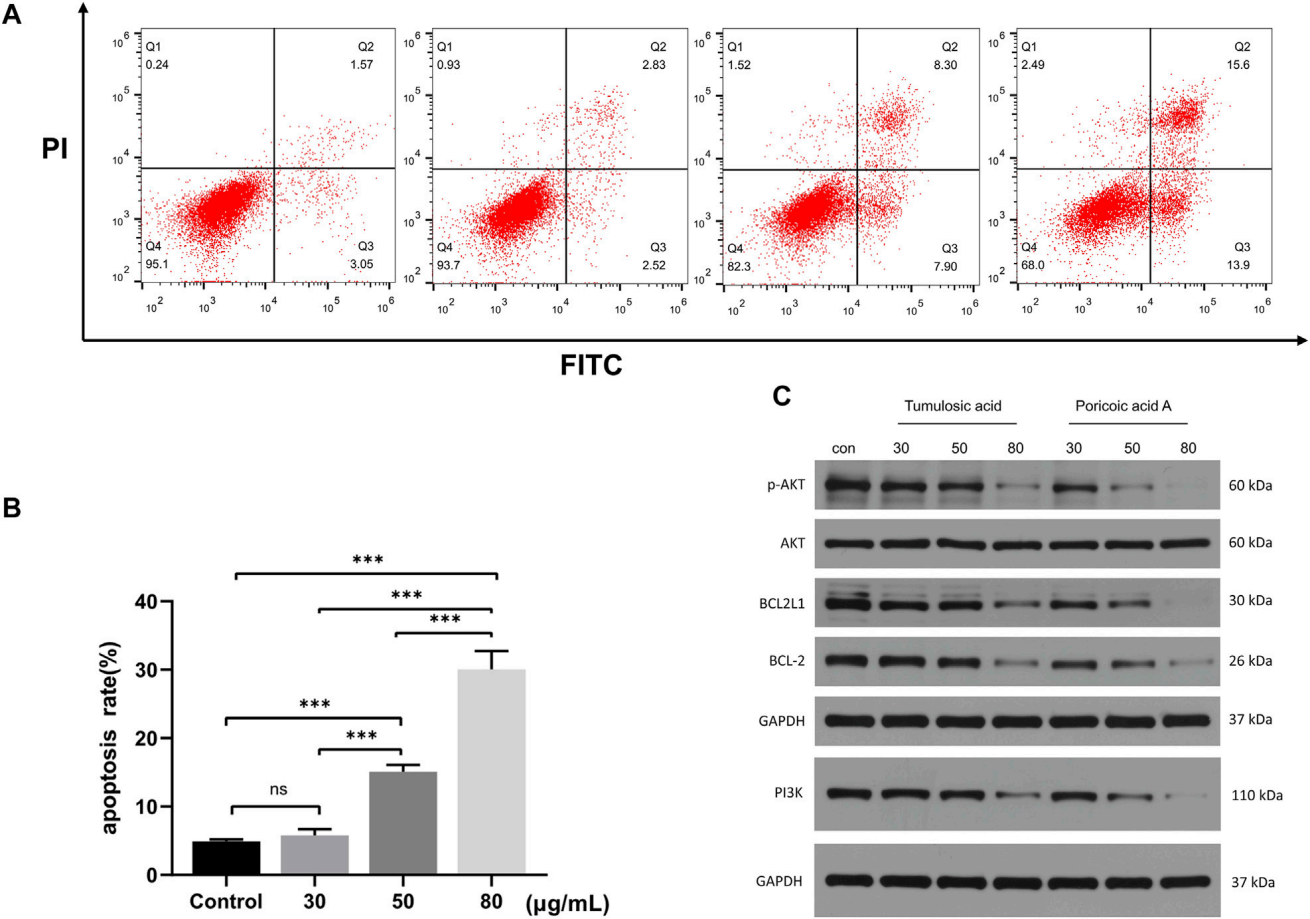
**(A)** SKOV3 cells were treated with various concentrations tumulosic acid. Apoptosis was analyzed by flow cytometry after Annexin V-FITC/PI staining. **(B)** The percentage of apoptotic cells was presented as the mean ± SD of three independent experiments, ***p < 0.01. **(C)** Tumulosic acid or poricoic acid A inhibits the PI3K/AKT pathway in OC cells. Representative Western blots showing the status of PI3K, AKT, *p*-AKT, BCL2, BCL2L1 in SKOV3.

The percentage of apoptotic cells increased with the increasing concentration of tumulosic acid, and the differences between groups were statistically significant (Figures 9A,B), strongly indicating that tumulosic acid induces apoptosis in SKOV3 cells.

#### Tumulosic Acid or Poricoic Acid a Inhibits the PI3K/AKT Pathway in SKOV3 Cells

The PI3K/AKT pathway is a key pathway for tumulosic acid or poricoic acid A against OC. It was found by Western blotting that tumulosic acid or poricoic acid A could affect the expression of key proteins in the pathway. As shown in Figure 9B, the protein levels of PI3K, *p*-AKT, BCL2L1 and BCL2 were significantly decreased in a drug dose-dependent manner. These results indicated that tumulosic acid or poricoic acid A could induce apoptosis in SKOV3 cells.

## Discussion

OC is a disease characterized by a late-stage presentation and poor prognosis (Moufarrij et al., 2019). Aggressive chemotherapy becomes an important treatment modality. However, recurrence rates gradually increased in chemotherapy patients, and the severe side effects also have a huge impact on patients’ lives (Lheureux et al., 2019b). Thus, the search for complementary therapies has become the task of many clinical studies in recent years. Evidence is emerging that *Poria cocos* compounds can be used as adjunctive therapy to improve patient symptoms (Xiangyan 2017), but it is also controversial. We therefore collected data from all published RCTs for a rigorously designed meta-analysis to assess the efficacy and safety of Chinese herbal medicine in OC treatment. Besides, the complex composition of *Poria cocos* and the novel mechanism of action against OC have greatly hindered the clinical popularity of *Poria cocos*. Hence, we used network pharmacology, molecular docking and cell experiments to identify the main components and elucidate the potential mechanism.

We included a total of 13 studies, which, although small in number, are valuable in assessing the effectiveness of *Poria cocos* formulas in combination with chemotherapy in the treatment of OC. And according to our knowledge, this is the first systematic evaluation of the clinical efficacy of *Poria cocos* formulas combined with paclitaxel-carboplatin in OC. In the current study, TRR, TCM syndrome score, KPS, QOL, and side effects subjective outcome indicators were used to evaluate the efficacy of the compounds, which was the same as the previous evaluation of traditional Chinese medicine (Li Y. et al., 2020). The results found that herbal medicine could significantly improve the TRR of patients. This is consistent with previous experimental studies that *Poria cocos* combined with chemotherapeutic drugs can effectively inhibit the proliferation and migration of cancer cells, hinder tumor progression, and improve the overall efficiency of treatment (Wang et al., 2018; Li et al., 2019). It has been shown that some Chinese herbal compounds can significantly improve the KPS score and TCM syndrome score of cancer patients, which has critical clinical values (He et al., 2021). Based on this, our meta-analysis found the same effect of *Poria cocos* compounds for OC. However, the effect of *Poria cocos* compounds combined with chemotherapy on patients’ quality of life is controversial. Several studies have found that *Poria cocos* compounds can effectively improve physical, cognitive, emotional, and social function (Ruojia 2013; Xiangyan 2017; Fangfang 2019), but one study did not support this finding (Chan et al., 2011). Our meta-analysis found that *Poria cocos* formulas combined with chemotherapy could significantly improve patients quality of life, physical function, and social function. However, for emotional function and cognitive function, there was no statistical significance. We detected differences in treatment duration and clinical stage in the included studies, which may account for the high heterogeneity. Due to the comparatively small sample sizes of the studies enrolled in our analysis, we cannot identify the possibility that the *Poria cocos* compounds can improve all functions, so more clinical studies are needed to confirm this further. The adverse effects of *Poria cocos* formulas combined with paclitaxel-carboplatin versus paclitaxel-carboplatin alone were evaluated and compared comprehensively, and the results showed that *Poria* compounds in combination with paclitaxel-carboplatin were more effective and safer.

**FIGURE 10:**
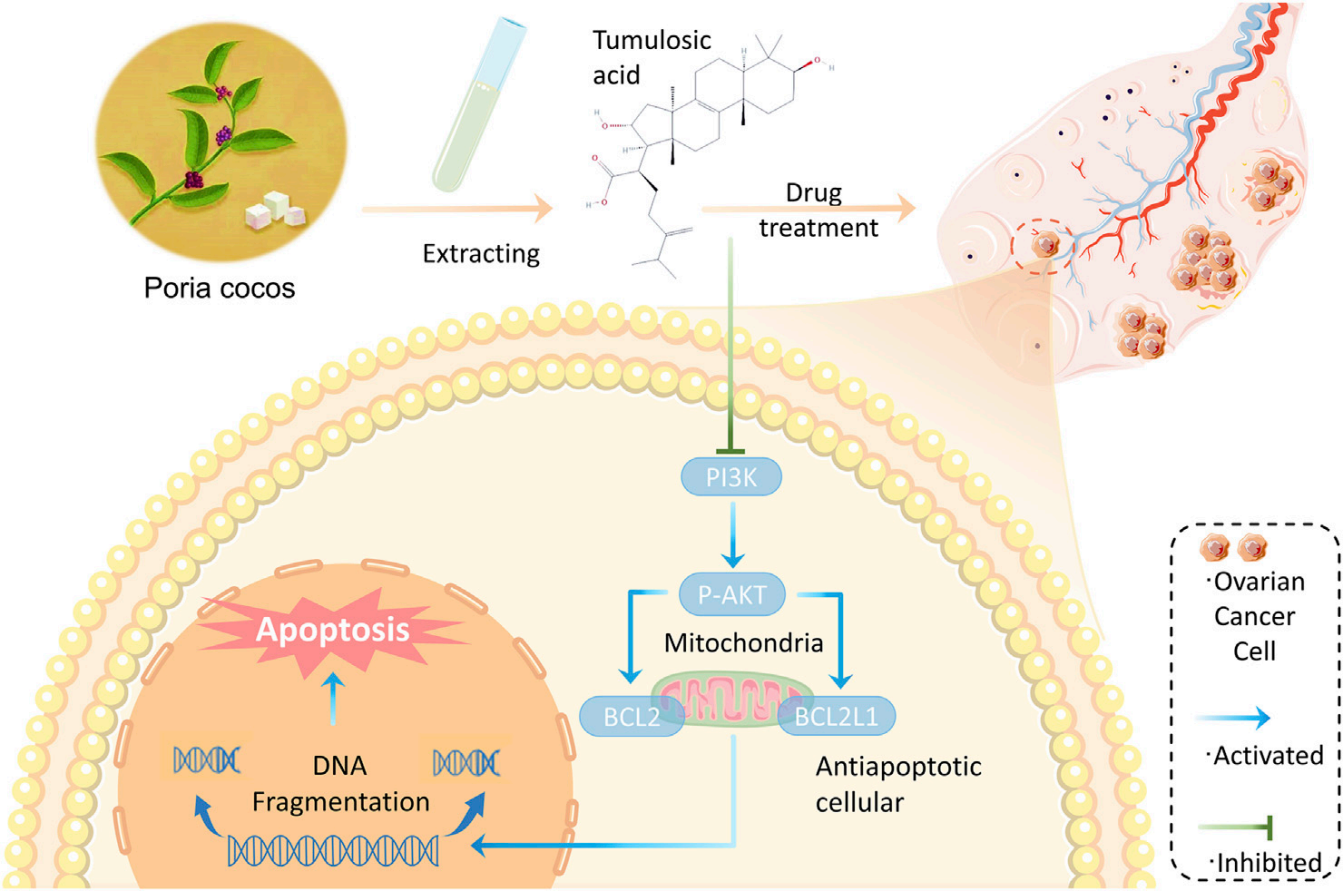
Schematic diagram of tumulosic acid acting on PI3K/AKT signaling pathway to induce apoptosis of SKOV3 cells. Combining the network pharmacology analysis and our results, we hypothesized that tumulosic acid influences the PI3K/AKT signaling pathway to regulate the expression of BCL2L1 to induce apoptosis in ovarian cancer cells.

*Poria cocos* contains two main bioactive components: triterpene acids and polysaccharides. Terpenoids are usually considered to be the main chemical and pharmacologically active components of *Poria cocos*, such as poricoic acid A (Nie et al., 2020). While β-glucan is considered to be the main polysaccharide in *Poria cocos* (Jin et al., 2003). Numerous studies have shown that these active ingredients have anti-tumor (Sun and Xia 2018), anti-inflammatory (Lee et al., 2017), antioxidant (Wang et al., 2016) and immunomodulatory effects (Pu et al., 2019). Through further network topology analysis, some important active components such as tumulosic acid, poricoic acid A, and some key targets such as BCL2L1, STAT3, and CASP3 have been identified. In addition, the functional enrichment analysis of core targets indicated that the anti-OC effect of *Poria cocos* might be related to PI3K-Akt signaling pathway. tumulosic acid ((2R)-2-[(3S,5R,10S,13,14,16, 17R)-3,16-dihydroxy-4,4,10,13,14-pentamethyl-2,3,5,6,7,11, 12, 15,16,17-decahydro-1H-cyclopenta [a]phenanthren-17-yl]-6-methyl-5-methylideneheptanoic acid) is a novel, less frequently reported compound with oral bioavailability and drug-like properties of 29.88% and 0.81, respectively. Previous research has established that compounds with oral bioavailability ≥30% and drug-likeness ≥ 0.18 can become effective drugs (Li L. et al., 2020; Li H. et al., 2020). Furthermore, based on the molecular docking results, tumulosic acid showed good binding activity to key targets, indicating that acid is likely to be the key active ingredient of *Poria cocos* against OC. BCL2L1 is a critical anti-apoptotic gene encoding the anti-apoptotic protein BCL-xL, an important transmembrane molecule of mitochondria that is closely associated with OC progression (Kurita et al., 2012; Guo et al., 2021). Interestingly, the differential expression of BCL2L1 in normal and OC tissues and the survival analysis results likewise provide strong arguments for this contention (Figure 7). Increasing evidence indicates that the PI3K/AKT pathway plays an important role in cancer development. It was reported that MiR-200b-5p could inhibit OC cell proliferation and promote apoptosis by targeting ATAD2 expression and regulating PI3K/AKT signaling pathway (Wang et al., 2020). Another study showed that MicroRNA-489 targeting XIAP inhibits the biological progress of cancer by regulating PI3K/AKT signaling and epithelial-mesenchymal transition (Jiang et al., 2020). Furthermore, a recent study showed that overexpression of the long-chain non-coding RNA H19 downregulated miR-140–5p and activated the PI3K/AKT signaling pathway to promote OC cell invasion, migration, and epithelial-mesenchymal transition (Xu et al., 2021). Previous studies have found that poricoic acid A, the main component of *Poria cocos*, can induce apoptosis in ovarian cancer cells by regulating the mTOR/p70s6k signaling axis (Ma et al., 2021). And our research has identified another important active component of *Poria cocos*, so we want to further explore the anticancer effect of tumulosic acid, and compare the effects of poricoic acid A with tumulosic acid. The results found that tumulosic acid can also induce apoptosis of cancer cells and exert its effect by regulating the PI3K-Akt signaling pathway. Additionally, there have been no reports regarding tumulosic acid induces apoptosis through acting on the PI3K/AKT pathway to reduce the expression of BCL2L1 in OC (Figure 10).

Our study has several limitations. We included small RCTs in China, which may have led to some bias in the results. We expect more high-quality RCTs to be published in international journals in the future to provide a theoretical basis for the subsequent clinical application of *Poria cocos* compounds. In addition, the dosage of the compound has not been standardized. For TRR, TCM Syndrome Score, KPS, and QOL, we included fewer than ten studies and did not assess publication bias for these groups.

## Conclusion

The meta-analysis of this research provides evidence to support the efficacy and safety of *Poria cocos* formulas in combination with paclitaxel-carboplatin in the treatment of OC, and further network pharmacology, molecular docking and vitro validation elucidate the underlying mechanisms. The findings of this study can contribute to the clinical evidence and theoretical basis for the combination of *Poria cocos* formulas with chemotherapy for OC. This integrated pharmacological strategy proposed in our study provides an excellent example for the mechanistic study of complex formulas.

## Data Availability

The original contributions presented in the study are included in the article/Supplementary Material, further inquiries can be directed to the corresponding author.

## Abbreviations

BP, Biological processes; CC, Cellular component; CI, Confidence intervals; CNKI, China Academic Journals; CPTAC, Clinical Proteomic Tumor Analysis Consortium; CR, Complete response; ETCM, Encyclopedia of Traditional Chinese Medicine; GO, Gene ontology; KEGG, Kyoto Encyclopedia of Genes and Genomes; KPS, Karnofsky Performance Scale; MD, Mean difference; MF, Molecular function; OC, Ovarian cancer; OTP, Open Targets Platform; PBS, Phosphate-buffered saline; PD, Progressive disease; PI, Proportional Integral; PPI, Protein-protein interaction; PR, Partial response; QOL, Quality of life; RR, Risk ratio; SD, Stable disease; SMD, Standardized mean difference; TCM, Traditional Chinese medicine; TCMSP, Traditional Chinese Medicine database and Analysis Platform; TRR, Tumor response rate; V-FITC/PI, V-fluorescein isothiocyanate/propidium iodide.
