# Supplementary figures and images for: Efficacy and Pharmacological Mechanism of Poria cocos-Based Formulas Combined With Chemotherapy for Ovarian Cancer: A Integrated Systems Pharmacology Study
Source: Front Pharmacol. 2022 Mar 21;13:788810. doi: 10.3389/fphar.2022.788810 (PMC8985862; doi:10.3389/fphar.2022.788810)

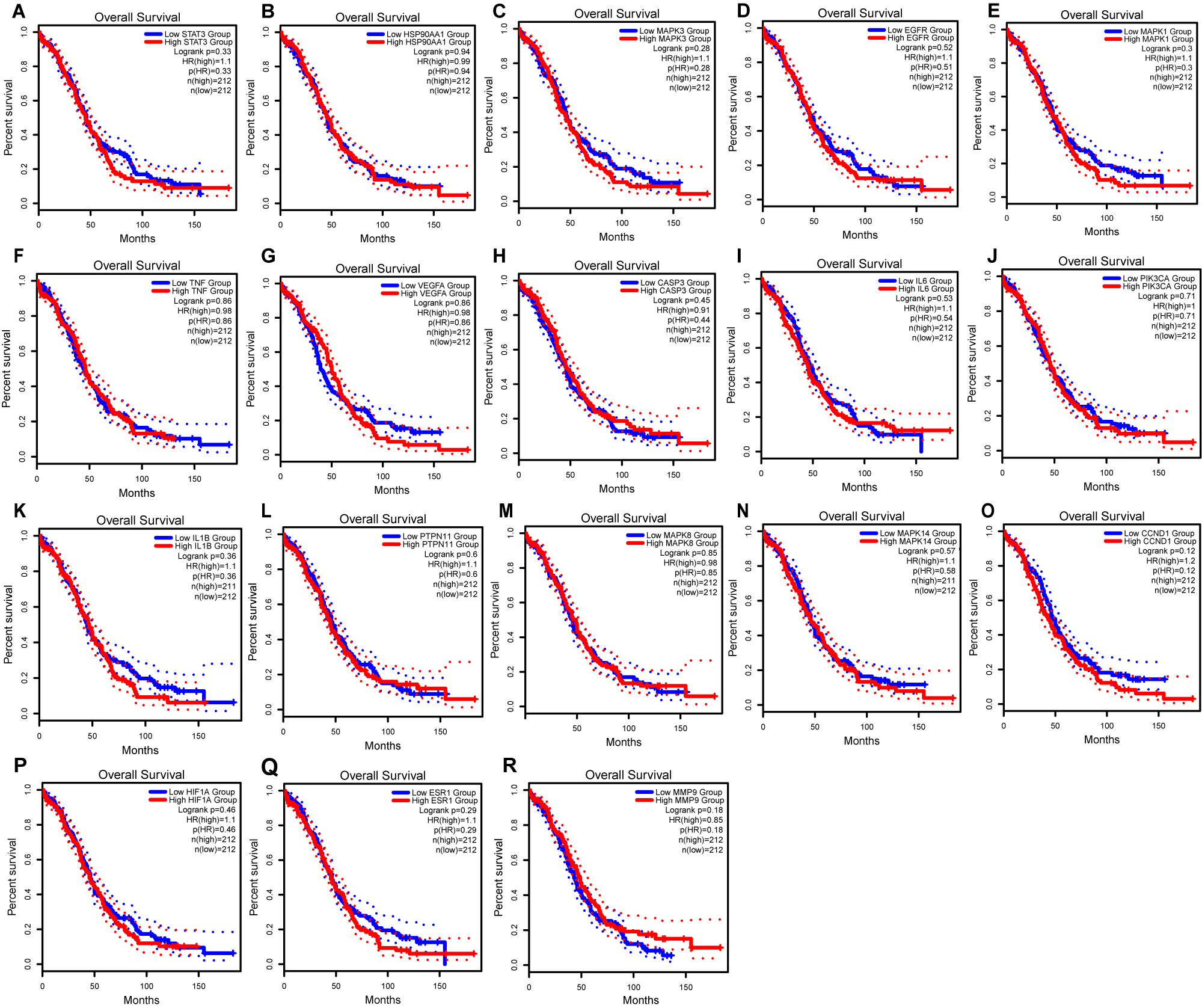

Supplement: Supplementary file 3 [file Image3.TIF]

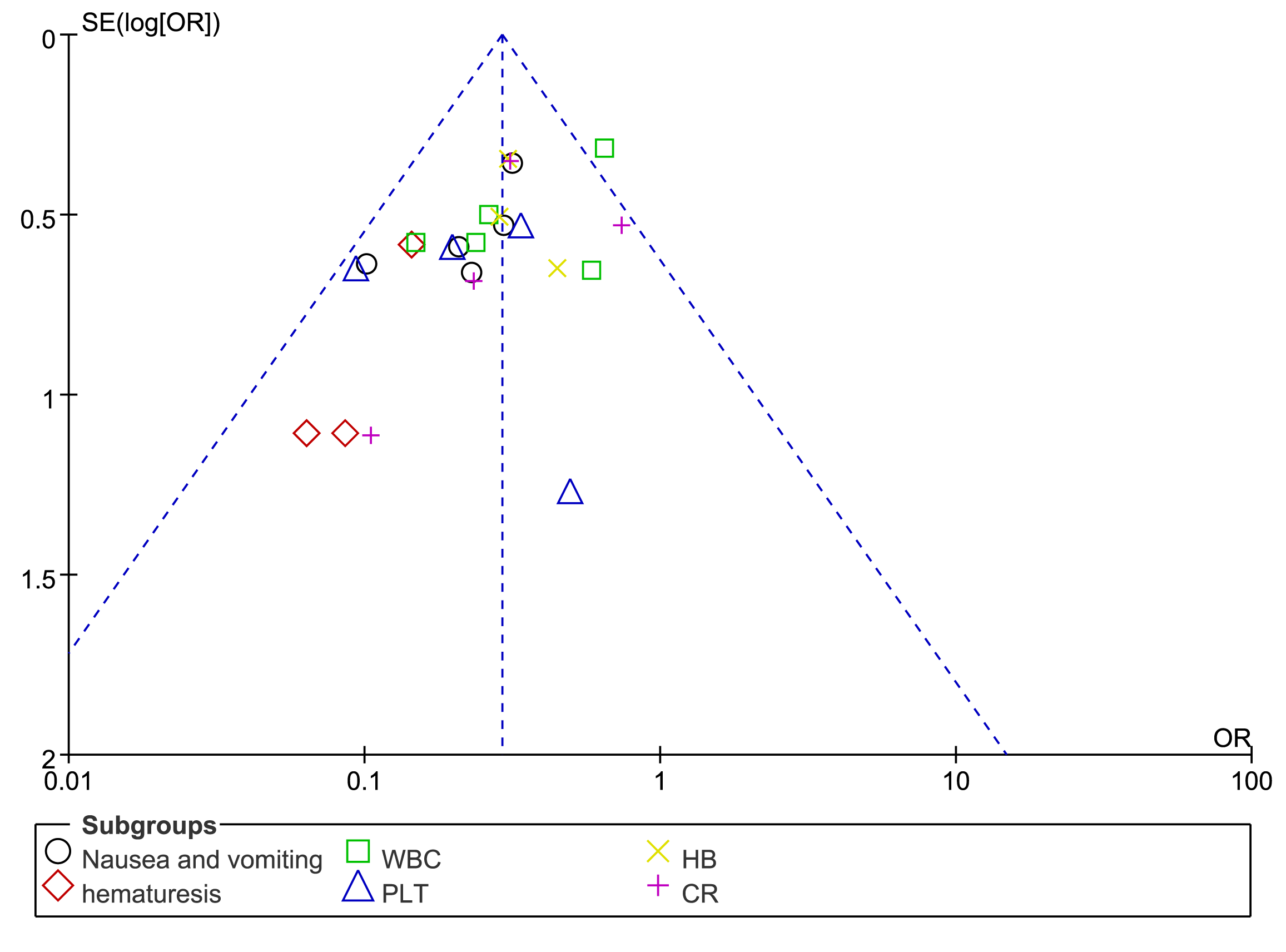

Supplement: Supplementary file 4 [file Image2.TIF]

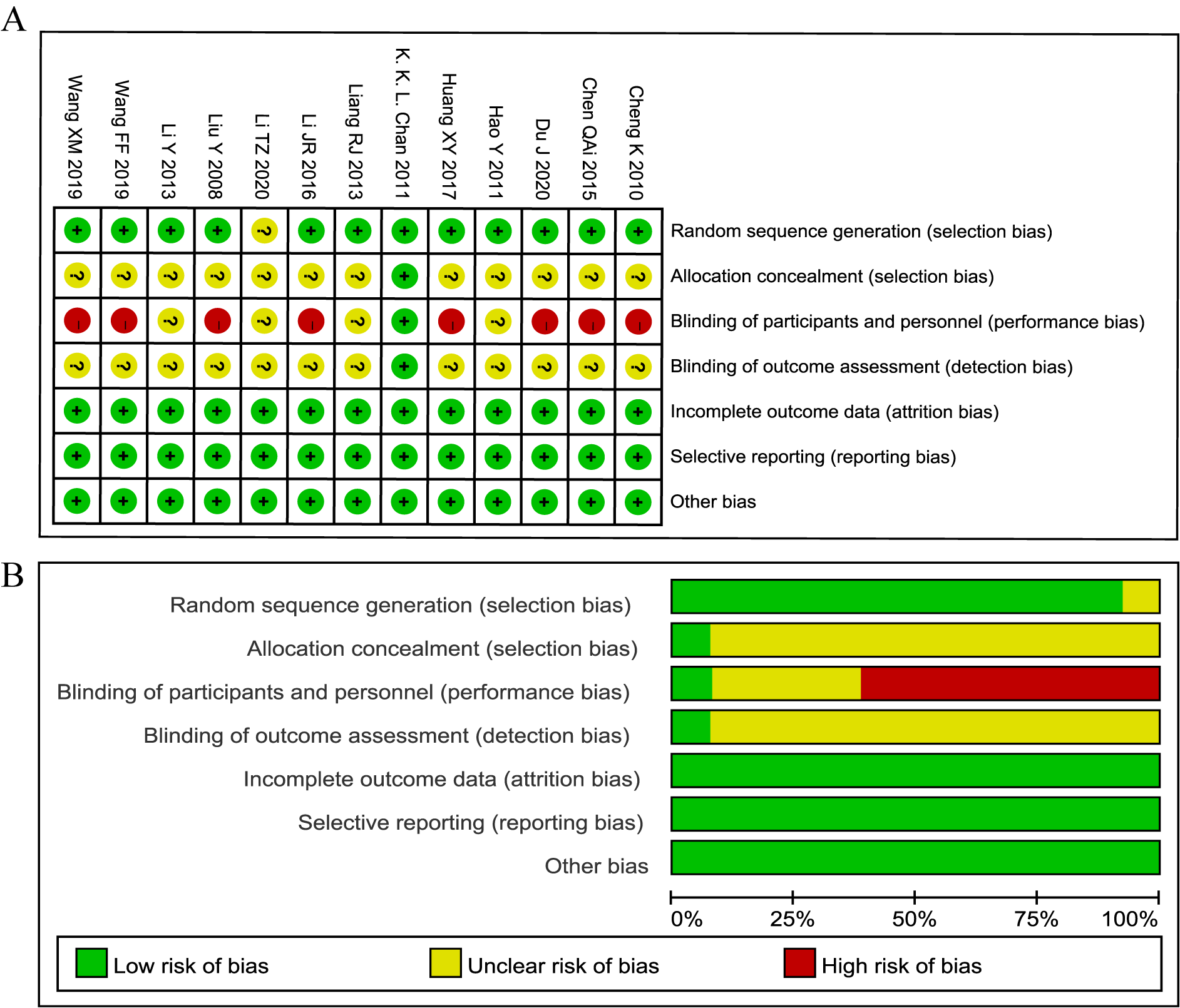

Supplement: Supplementary file 5 [file Image1.TIF]
