# Supplementary material for: Efficacy and Pharmacological Mechanism of Poria cocos-Based Formulas Combined With Chemotherapy for Ovarian Cancer: A Integrated Systems Pharmacology Study
Source: Front Pharmacol. 2022 Mar 21;13:788810. doi: 10.3389/fphar.2022.788810 (PMC8985862; doi:10.3389/fphar.2022.788810)
Supplement: Supplementary file 6 [file Table5.DOCX]

Search Strategy

Pubmed

The detailed search strategy used in PubMed was: ((("Wolfiporia"[Mesh]) OR ((Wolfiporia extensa[Title/Abstract]) OR (Poria cocos[Title/Abstract]) OR (Tuckahoe Fungus[Title/Abstract]) OR (Fungus, Tuckahoe[Title/Abstract]) OR (Fu-Ling[Title/Abstract]) OR (Fu Ling[Title/Abstract]) OR (Wolfiporia cocos[Title/Abstract]) OR (Fuling[Title/Abstract]))) OR (("Medicine, Chinese Traditional"[Mesh]) OR ((Traditional Chinese Medicine[Title/Abstract]) OR (Chung I Hsueh[Title/Abstract]) OR (Hsueh, Chung I[Title/Abstract]) OR (Traditional Medicine, Chinese[Title/Abstract]) OR (Zhong Yi Xue[Title/Abstract]) OR (Chinese Traditional Medicine[Title/Abstract]) OR (Chinese Medicine, Traditional[Title/Abstract]) OR (Traditional Tongue Diagnosis[Title/Abstract]) OR (Tongue Diagnoses, Traditional[Title/Abstract]) OR (Tongue Diagnosis, Traditional[Title/Abstract]) OR (Traditional Tongue Diagnoses[Title/Abstract]) OR (Traditional Tongue Assessment[Title/Abstract]) OR (Tongue Assessment, Traditional[Title/Abstract]) OR (Traditional Tongue Assessments[Title/Abstract])))) AND (("Ovarian Neoplasms"[Mesh]) OR ((Neoplasm, Ovarian[Title/Abstract]) OR (Ovarian Neoplasm[Title/Abstract]) OR (Ovary Neoplasms[Title/Abstract]) OR (Neoplasm, Ovary[Title/Abstract]) OR (Neoplasms, Ovary[Title/Abstract]) OR (Ovary Neoplasm[Title/Abstract]) OR (Neoplasms, Ovarian[Title/Abstract]) OR (Ovary Cancer[Title/Abstract]) OR (Cancer, Ovary[Title/Abstract]) OR (Cancers, Ovary[Title/Abstract]) OR (Ovary Cancers[Title/Abstract]) OR (Ovarian Cancer[Title/Abstract]) OR (Cancer, Ovarian[Title/Abstract]) OR (Cancers, Ovarian[Title/Abstract]) OR (Ovarian Cancers[Title/Abstract]) OR (Cancer of Ovary[Title/Abstract]) OR (Cancer of the Ovary[Title/Abstract])))

The search date: 20 July 2021

The number of the results: 76

Web of Science

The detailed search strategy used in Web of Science was:

#1 (TS=(Wolfiporia OR Wolfiporia extensa OR Poria cocos OR Tuckahoe Fungus OR Fungus, Tuckahoe OR Fu-Ling OR Fu Ling OR Wolfiporia cocos OR Fuling OR Medicine, Chinese Traditional OR Traditional Chinese Medicine OR Chung I Hsueh OR Hsueh, Chung I OR Traditional Medicine, Chinese OR Zhong Yi Xue OR Chinese Traditional Medicine OR Chinese Medicine, Traditional OR Traditional Tongue Diagnosis OR Tongue Diagnoses, Traditional OR Tongue Diagnosis, Traditional OR Traditional Tongue Diagnoses OR Traditional Tongue Assessment OR Tongue Assessment, Traditional OR Traditional Tongue Assessments)) AND LA=(English)

#2 (TS=(Ovarian Cancer OR Ovarian Neoplasms OR Neoplasm, Ovarian OR Ovarian Neoplasm OR Ovary Neoplasms OR Neoplasm, Ovary OR Neoplasms, Ovary OR Ovary Neoplasm OR Neoplasms, Ovarian OR Ovary Cancer OR Cancer, Ovary OR Cancers, Ovary OR Ovary Cancers OR Ovarian Cancer OR Cancer, Ovarian OR Cancers, Ovarian OR Ovarian Cancers OR Cancer of Ovary)) AND LA=(English)

#3 #1 AND #2

The search date: 20 July 2021

The number of the results: 172

Embase

The detailed search strategy used in Embase was:

#1,"'ovary cancer'/exp"

#2,"'ovarian neoplasms':ab,ti OR 'neoplasm, ovarian':ab,ti OR 'ovarian neoplasm':ab,ti OR 'ovary neoplasms':ab,ti OR 'neoplasm, ovary':ab,ti OR 'neoplasms, ovary':ab,ti OR 'ovary neoplasm':ab,ti OR 'neoplasms, ovarian':ab,ti OR 'cancer, ovary':ab,ti OR 'cancers, ovary':ab,ti OR 'ovary cancers':ab,ti OR 'ovarian cancer':ab,ti OR 'cancer, ovarian':ab,ti OR 'cancers, ovarian':ab,ti OR 'ovarian cancers':ab,ti OR 'cancer of ovary':ab,ti OR 'cancer of the ovary':ab,ti"

#3,"#1 OR #2"

#4,"'wolfiporia cocos'/exp"

#5,"'wolfiporia extensa':ab,ti OR 'poria cocos':ab,ti OR 'tuckahoe fungus':ab,ti OR 'fungus, tuckahoe':ab,ti OR 'fu-ling':ab,ti OR 'fu ling':ab,ti OR 'wolfiporia':ab,ti OR 'fuling':ab,ti

#6,"' medicine, chinese traditional '/exp"

#7,"'traditional chinese medicine':ab,ti OR 'chung i hsueh':ab,ti OR 'hsueh, chung i':ab,ti OR 'traditional medicine, chinese':ab,ti OR 'zhong yi xue':ab,ti OR 'chinese traditional medicine':ab,ti OR 'chinese medicine, traditional':ab,ti OR 'traditional tongue diagnosis':ab,ti OR 'tongue diagnoses, traditional':ab,ti OR 'tongue diagnosis, traditional':ab,ti OR 'traditional tongue diagnoses':ab,ti OR 'traditional tongue assessment':ab,ti OR 'tongue assessment, traditional':ab,ti OR 'traditional tongue assessments':ab,ti"

#8,"#4 OR #5 OR #6 OR #7"

#9,"#3 AND #8"

The search date: 23 July 2021

The number of the results: 135

Cochrane Library

#1 MeSH descriptor: [Ovarian Neoplasms] explode all trees

#2 (Neoplasm, Ovarian):ti,ab,kw OR (Ovarian Neoplasm):ti,ab,kw OR (Ovary Neoplasms):ti,ab,kw OR (Neoplasm, Ovary):ti,ab,kw OR (Neoplasms, Ovary):ti,ab,kw OR (Ovary Neoplasm):ti,ab,kw OR (Neoplasms, Ovarian):ti,ab,kw OR (Ovary Cancer):ti,ab,kw OR (Cancer, Ovary):ti,ab,kw OR (Cancers, Ovary):ti,ab,kw OR (Ovary Cancers):ti,ab,kw OR (Ovarian Cancer):ti,ab,kw OR (Cancer, Ovarian):ti,ab,kw OR (Cancers, Ovarian):ti,ab,kw OR (Ovarian Cancers):ti,ab,kw OR (Cancer of Ovary):ti,ab,kw OR (Cancer of the Ovary):ti,ab,kw

#3 #1 OR #2

#4 MeSH descriptor: [Wolfiporia] explode all trees

#5 (Wolfiporia extensa):ti,ab,kw OR (Poria cocos):ti,ab,kw OR (Tuckahoe Fungus):ti,ab,kw OR (Fungus, Tuckahoe):ti,ab,kw OR (Fu-Ling):ti,ab,kw OR (Fu Ling):ti,ab,kw OR (Wolfiporia cocos):ti,ab,kw OR (Fuling):ti,ab,kw

#6 MeSH descriptor: [Medicine, Chinese Traditional] explode all trees

#7 (Traditional Chinese Medicine):ti,ab,kw OR (Chung I Hsueh):ti,ab,kw OR (Hsueh, Chung I):ti,ab,kw OR (Traditional Medicine, Chinese):ti,ab,kw OR (Zhong Yi Xue):ti,ab,kw OR (Chinese Traditional Medicine):ti,ab,kw OR (Chinese Medicine, Traditional):ti,ab,kw OR (Traditional Tongue Diagnosis):ti,ab,kw OR (Tongue Diagnoses, Traditional):ti,ab,kw OR (Tongue Diagnosis, Traditional):ti,ab,kw OR (Traditional Tongue Diagnoses):ti,ab,kw OR (Traditional Tongue Assessment):ti,ab,kw OR (Tongue Assessment, Traditional):ti,ab,kw OR (Traditional Tongue Assessments):ti,ab,kw

#8 #4 OR #5 OR #6 OR #7

#7 #3 AND #8

The search date: 23 July 2021

The number of the results: 14

China Academic Journals (CNKI)

(Thematic = Fuling or Thematic =Traditional Chinese Medicine or Thematic =

Chinese herb) and (Thematic = Ovarian cancer or Thematic = Ovarian tumor) (Exact match) The search date: 23 July 2021

The number of the results: 393

Chinese Science and Technology Journals (CQVIP)

(Title or keywords = “Fuling” or “Traditional Chinese Medicine” or“Chinese herb”) and (Title or keywords = “Ovarian cancer” or “Ovarian tumor”)

The search date: 23 July 2021

The number of the results: 193

Wanfang

Title or keywords: (“Fuling” or “Traditional Chinese Medicine” or“Chinese herb”) and Title or keywords: (“Ovarian cancer” or “Ovarian tumor”)

The search date: 23 July 2021

The number of the results: 793

Chinese Biomedical Literature database

#3 #1 AND #2

#2 " “Ovarian cancer” or “Ovarian tumor” "[ Title/Abstract]

#1 " “Fuling” or “Traditional Chinese Medicine” or“Chinese herb”"[ Title/Abstract]

The search date: 23 July 2021

The number of the results: 480
